# Composition Fluctuations in Lipid Bilayers

**DOI:** 10.1016/j.bpj.2017.10.009

**Published:** 2017-12-19

**Authors:** Svetlana Baoukina, Dmitri Rozmanov, D. Peter Tieleman

**Affiliations:** 1 Department of Biological Sciences and Centre for Molecular Simulation, University of Calgary, Calgary, Alberta, Canada; 2 Department of Information Technologies, University of Calgary, Calgary, Alberta, Canada

## Abstract

Cell membranes contain multiple lipid and protein components having heterogeneous in-plane (lateral) distribution. Nanoscale rafts are believed to play an important functional role, but their phase state—domains of coexisting phases or composition fluctuations—is unknown. As a step toward understanding lateral organization of cell membranes, we investigate the difference between nanoscale domains of coexisting phases and composition fluctuations in lipid bilayers. We simulate model lipid bilayers with the MARTINI coarse-grained force field on length scales of tens of nanometers and timescales of tens of microseconds. We use a binary and a ternary mixture: a saturated and an unsaturated lipid, or a saturated lipid, an unsaturated lipid, and cholesterol, respectively. In these mixtures, the phase behavior can be tuned from a mixed state to a coexistence of a liquid-crystalline and a gel, or a liquid-ordered and a liquid-disordered phase. Transition from a two-phase to a one-phase state is achieved by raising the temperature and adding a hybrid lipid (with a saturated and an unsaturated chain). We analyze the evolution of bilayer properties along this transition: domains of two phases transform to fluctuations with local ordering and compositional demixing. Nanoscale domains and fluctuations differ in several properties, including interleaflet overlap and boundary length. Hybrid lipids show no enrichment at the boundary, but decrease the difference between the coexisting phases by ordering the disordered phase, which could explain their role in cell membranes.

## Introduction

Lipid bilayers constitute the basis for cell membranes. They contain multiple lipid species, which are distributed nonuniformly along the bilayer plane (1). This distribution modulates the bilayer properties and creates an optimized environment for protein function. The raft hypothesis suggests that membrane components exhibit nanoscale dynamic organization (2). Rafts are enriched in saturated lipids and cholesterol and incorporate selected proteins (3). They have sizes in the range of 10–200 nm and short lifetimes (10^−3^–10° s). Rafts are important in membrane trafficking, signal transduction, and entry of pathogens (4, 5, 6, 7). However, direct experimental observation of rafts is challenging due to their dynamic nanoscale nature. Rafts are believed to constitute nanodomains of the liquid-ordered (Lo) phase in the liquid-disordered (Ld) phase. Yet the small size and short lifetime of such Lo domains cannot be explained by classical theories for phase separation. With limited in vivo evidence of the existence of rafts (8, 9, 10, 11), their phase state and underlying mechanisms remain elusive (12, 13).

The difficulty in characterizing the lateral organization of cell membranes lies in their complexity. A crowded environment with many lipid and protein players, it is coupled to the cytoskeleton and affected by active cellular processes (14). Characterizing phase behavior of lipids alone requires building multidimensional phase diagrams (15). Numerous membrane proteins interact preferentially with different lipids, and vary in size, membrane partitioning, and mobility. Lipid transport interferes kinetically with these interactions, and coupling to cytoskeleton bounds them spatially. Many theories describing these phenomena attempt to explain raft formation (for review, see (14, 16, 17)). Yet distinguishing between these different theories is difficult, in part due to a lack of understanding of lipid phase behavior.

Lipid-lipid interactions alone could produce rafts via several different mechanisms (18). The first group of mechanisms is based on phase separation with limited growth of domains. Domain growth could be limited due to interdomain repulsion. Repulsion may be caused by uncompensated headgroup dipoles in the domains (19) or by domain curvature (20). A large number of small domains could become favorable due to entropy gain at low line tension at phase boundaries (21). Line tension may be lowered by linactants, for example by hybrid lipids. The second group of mechanisms is based on dynamic heterogeneity with local structure and order (22). Dynamic heterogeneity develops in one phase due to lateral density and composition fluctuations. Fluctuations increase in magnitude upon approaching a phase transition and become extremely strong in the vicinity of a critical point (23). Rafts could be a manifestation of 2D microemulsion—a liquid with local structure and a tendency to order (24).

Substantial progress in understanding lipid phase behavior has been achieved by studying lipid bilayers of simple composition. The coexistence of macroscopic domains of the Lo and Ld phases has been experimentally reproduced in mixtures of saturated and unsaturated lipids and cholesterol (for review, see (25, 26)). Emerging techniques provided details on the properties of coexisting Lo and Ld phases (27, 28). Transition from macro- to nanoscale Lo domains has been observed upon substitution of unsaturated lipids by so-called hybrid lipids with one saturated and one unsaturated chain (29, 30). However, the properties of nanodomains are measured indirectly, and it is difficult to establish their phase state. Due to limits in achievable spatial and temporal resolution, distinguishing between nanoscale domains and fluctuations in experiments remains a challenging task.

Here, we investigate the differences between nanoscale domains of coexisting phases and composition fluctuations in lipid bilayers as a step toward understanding lateral organization of cell membranes. We simulate model lipid bilayers above and below their miscibility transition temperatures with the Martini coarse-grained force field (31). The Martini force field has been widely used to study the properties of lipid bilayers of simple composition, and, in a recent study, of complex mixtures modeling the real plasma membranes (32). Phase separation into the Ld and Lo phases has been reproduced in a number of Martini simulations (for review, see (33, 34)); the driving forces for phase separation and the limitations of the Martini model have been discussed (35, 36, 37). In this work, we focus on the transition between domains of coexisting phases and composition fluctuations in lipid bilayers. This topic, to our knowledge, has not been previously studied in simulations. We consider two types of phase coexistence relevant for cell membranes (liquid-liquid and liquid-solid), and change the bilayer phase behavior by varying the temperature and adding a hybrid lipid. We observe that hybrid lipids are not enriched at the phase boundary, but increase the order of the disordered domains, thus decreasing the difference between the coexisting phases. This suggests a potential biological role of tuning the size and order of domains in cell membranes. We find that composition fluctuations and domains of coexisting phases differ in several properties, including interleaflet overlap and boundary length. These properties could be used to distinguish nanoscale domains from fluctuations in experiments, and to obtain insights on the nature of rafts in cell membranes.

## Methods

We performed molecular dynamics simulations with the GROMACS software package (v.4.6.5) (38). Lipid bilayers were simulated with the Martini coarse-grained (CG) force field v. 2.1 (31). We used a binary mixture of dipalmitoyl-phosphatidylcholine (DPPC) and dilinoleoyl-phosphatidylcholine (DUPC) to model the coexistence of the liquid-crystalline (L*α*) and gel (L*β*) phases, and a ternary mixture of DPPC, DUPC, and cholesterol to model the coexistence of the Ld and Lo phases. The molar ratios for the two mixtures were 3:2, and 7:7:6, respectively. These mixtures were simulated in a temperature range of 270–340 K. At higher temperatures, the bilayers mixed and formed a single phase, in which the composition fluctuations were studied. At lower temperatures, the bilayers demixed and separated into domains of coexisting phases. A so-called hybrid lipid with one saturated and one unsaturated chain, palmitoyl-linoleoyl-phosphatidylcholine (PUPC), was added to the two mixtures in the following way: a specific fraction of the DUPC lipids in each leaflet was randomly replaced by the PUPC lipid, and the bilayer was equilibrated at a *T* = 340 K. The resulting molar ratios were DUPC/PUPC 8:2, 7:3, and 6:4 in the L*α*-gel mixture (20, 30, and 40% PUPC substituting DUPC), and 6:1, 5:2, and 4:3 in the Ld-Lo mixture (14, 29, and 43% PUPC substituting DUPC). These concentrations of the hybrid lipid were selected to achieve noticeable effects on phase separation and composition fluctuations, and, at the same time, to maintain phase coexistence in the selected interval of temperatures.

In the Martini force field, molecules are represented by particles that group approximately four heavy atoms together. All the lipids are standard components of the force field. The system setup consisted of a randomly mixed bilayer in water. Two system sizes were used: smaller bilayers (∼35 × 35 × 15 nm^3^) contained 4608 lipids and were solvated in 128,000 CG water particles; larger bilayers (∼70 × 70 × 30 nm^3^) contained 18,432 lipids and were solvated in 1,024,000 CG water particles. At lower temperatures (<290 K), the antifreeze water particles substituted ∼5% of regular water particles to prevent water crystallization, which is a standard practice in the Martini force field (31). For nonbonded interactions, the standard cutoffs for the Martini force field were used: the Lennard-Jones potential was shifted to 0 between 0.9 and 1.2 nm; the Coulomb potential was shifted to 0 between 0 and 1.2 nm with a relative dielectric constant of 15. The time step was 20 fs with neighbor-list updates every 10 steps. Lipids and water were coupled separately to a target temperature using the velocity rescaling thermostat (39) with a time constant of 1 ps. The normal and tangential pressures of 1 bar were maintained using the Berendsen barostat (40) with the semiisotropic coupling scheme, a time constant of 4 ps, and compressibility 5 ⋅ 10^−5^ bar^−1^. The simulation time was 10–30 *μ*s; longer times corresponded to the cases near or with phase separation. The last microsecond of the trajectory was used for analysis.

Quantitative analysis of composition fluctuations and domains of coexisting phases was performed using a combination of custom scripts. The areas per lipid were defined based on Voronoi tessellation using a MATLAB program (v. R2014b; The MathWorks, Natick, MA). Lateral heterogeneity was analyzed based on local lipid environment (41), defined as the first surrounding shell of nearest neighbors. The Voronoi sites with a local concentration of DPPC and cholesterol above the average concentration in the bilayer were assigned to an ordered cluster. The ordered clusters were then grouped together using the connectivity matrix. In these grouped clusters, the distinction between composition fluctuations and domains of coexisting phases was made based on structural and dynamic properties. The boundary was calculated as the sum of Voronoi edges between the cluster and its surroundings. The overlap (registration) of the clusters between the leaflets was calculated as the area of the clusters aligned (i.e., in register) in the two leaflets divided by to the total area of the clusters in the leaflet.

The in-plane 2D radial distribution function (RDF) for the bilayers was calculated as the average ratio of the lipid density at the distance *r* from the center of mass of the lipid molecule to the average density in the bilayer. In these calculations, we considered the unsaturated lipid only, as it is enriched in the disordered phase in all cases, without long-range translational order (which avoids additional undulations in RDFs corresponding to intermolecular distances). The correlation length, *ξ*, was calculated from an exponential fit to the 2D RDFs using the formula

(1) $$RDF\left(r\right)=a_{0}\cdot e^{-r/\xi }+a_{1}.$$

The correlation time, *τ*, was calculated from the time decay of the local density time correlation function using a single exponential fit as in (1). The local lipid density was sampled on a 2D grid of 20 × 20 cells from 10 consecutive trajectory time frames with the time step of 10 ps. It was assumed that such time is short relative to timescale of diffusion in the system so that the changes in the density are acceptably small.

The chain orientational order parameter, *S*_*z*_, was calculated using the formula

(2) $$S_{z,n}=\langle 1/2\left(3\cdot \cos^{2}\theta_{n}-1\right)\rangle ,$$

where *θ*_*n*_ is the angle between the vector connecting the *n*−1 and *n*+1 sites of the hydrocarbon chain and the monolayer normal *z* averaged over all sites for both chains and over all lipids, except for cholesterol.

2D density maps were calculated using the GROMACS g_densmap tool; the densities were averaged over varying time intervals of 100 ns, 1 and 9 *μ*s.

To calculate the membrane curvature, the membrane was fitted to a surface by interpolation using the GL particles in phospholipids and the ROH particle in cholesterol. The interpolated surface was placed on an equally spaced grid (0.4 nm) and smoothed using a binomial filter. This procedure was applied to each leaflet separately, and the obtained surface on a grid was averaged over the last microsecond of the trajectory. For the resulting surface, the partial derivatives were calculated to find the principal curvatures *c*_1_ and *c*_2_ as in previous studies (42). The mean curvature of the bilayer equals

(3) $$H=1/2\left(c_{1}+c_{2}\right).$$

The in-plane 2D static structure factor, $S\left(\bar{q}\right)$, was calculated from the scattering of the molecular centers of mass using the formula

(4) $$S\left(\bar{q}\right)=\frac{1}{N}{\left|\sum_{n}e^{-i\bar{q}\cdot {\bar{r}}_{n}}\right|}^{2}.$$

Here, the scattering length of all scattering centers, *N*, was assumed constant, $\bar{q}$ is the wave vector, and $\bar{r}$ is the real space vector. The calculated structure factor as a function of 2D wave vector was then averaged over the polar angle to give the radial component, *S*(*q*).

## Results

### Temperature dependence of the bilayer properties

**Figure 1:**
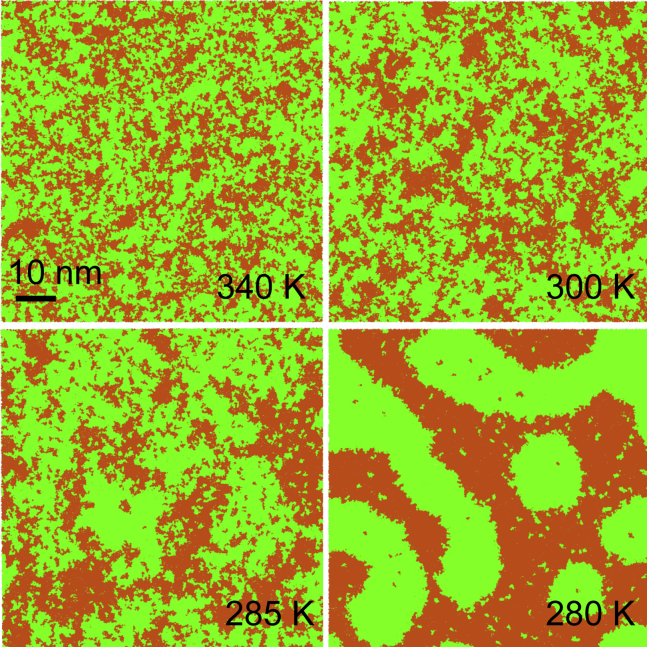
Phase behavior of the DPPC/DUPC 3:2 large bilayers at selected temperatures. View from top on the upper leaflet is given. DPPC is shown in green, DUPC in orange; water not shown. To see this figure in color, go online.

**Figure 2:**
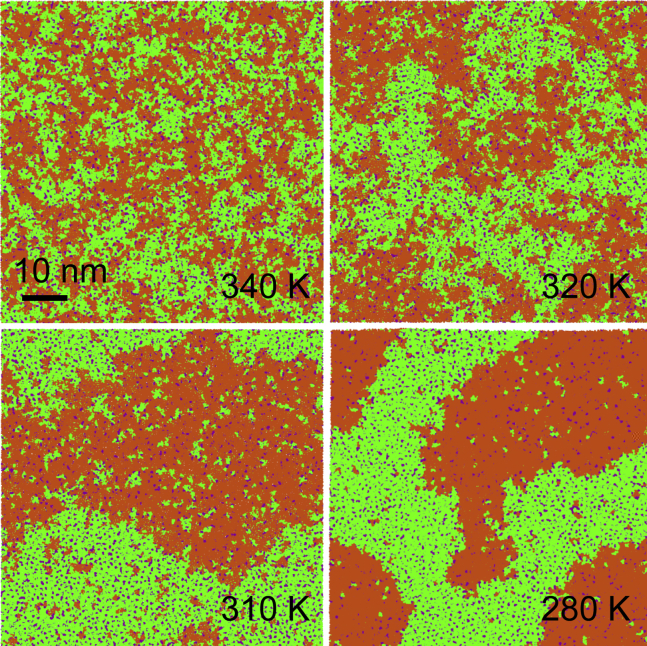
Phase behavior of the DPPC/DUPC/cholesterol 7:7:6 large bilayers at selected temperatures. View from top on the upper leaflet is given. DPPC is shown in green, DUPC in orange, cholesterol in purple; water not shown. To see this figure in color, go online.

We simulated lipid bilayers composed of mixtures of DPPC/DUPC in ratio 3:2 and of DPPC/DUPC/cholesterol in ratio 7:7:6. At lower temperatures, the bilayers separated into the coexisting L*α* and gel, and Ld and Lo phases, respectively. Upon raising the temperature, the bilayers mixed to form a single phase. Characteristic snapshots of the phase behavior in the two mixtures are shown in Figs. 1 and 2 and a summary of all simulations is given in Tables 1 and 2.

**Table 1:** The Properties of DPPC:DUPC 3:2 Bilayers

*T*,K	*A*_*L*_, nm^2^	*A*_*L*_,ordered nm^2^	*A*_*L*_,disord, nm^2^	*A*_DPPC_, nm^2^	*A*_DUPC_, nm^2^	*C*_DPPC_	*C*_DUPC_	*D*_DPPC_, 10^7^ cm^2^/s	*D*_DUPC_, 10^7^ cm^2^/s	*S*_*z*_	Phase
340	0.722 ± 0.002	0.682 ± 0.004	0.747 ± 0.003	0.674 ± 0.005	0.740 ± 0.013	0.88 ± 0.01	0.12 ± 0.01	4.1 ± 0.2	4.6 ± 0.1	0.30 ± 0.02	L*α*
320	0.692 ± 0.001	0.652 ± 0.004	0.719 ± 0.003	0.645 ± 0.004	0.712 ± 0.012	0.89 ± 0.01	0.11 ± 0.01	3.0 ± 0.1	3.1 ± 0.1	0.33 ± 0.03	L*α*
300	0.663 ± 0.001	0.621 ± 0.003	0.693 ± 0.003	0.614 ± 0.004	0.685 ± 0.009	0.91 ± 0.01	0.09 ± 0.01	1.7 ± 0.1	1.9 ± 0.2	0.35 ± 0.02	L*α*
290	0.648 ± 0.002	0.603 ± 0.003	0.686 ± 0.003	0.598 ± 0.003	0.665 ± 0.012	0.92 ± 0.01	0.08 ± 0.01	1.3 ± 0.1	1.4 ± 0.1	0.40 ± 0.04	L*α*
285	0.640 ± 0.001	0.593 ± 0.003	0.680 ± 0.003	0.587 ± 0.003	0.661 ± 0.012	0.93 ± 0.01	0.07 ± 0.01	1.0 ± 0.1	1.1 ± 0.1	0.42 ± 0.04	L*α*
280	0.569 ± 0.001	0.468 ± 0.001	0.690 ± 0.002	0.467 ± 0.001	0.565 ± 0.031	0.99 ± 0.01	0.01 ± 0.01	0.05 ± 0.01	0.7 ± 0.1	0.90 ± 0.01	Gel + L*α*
270	0.555 ± 0.001	0.464 ± 0.001	0.665 ± 0.002	0.463 ± 0.001	0.517 ± 0.019	0.99 ± 0.01	0.01 ± 0.01	0.02 ± 0.01	0.4 ± 0.1	0.91 ± 0.01	Gel + L*α*

Data for the small DPPC/DUPC 3:2 bilayer forming L*α*-gel phases. Here *A*_*L*_ is the average area per lipid in the bilayer; *A*_*L*_, ordered, in the ordered clusters; *A*_*L*_, disord, in the remaining disordered part; *A*_*i*_ values are the areas per lipid components in the ordered clusters; *C*_*i*_ values are the concentration of lipid components in the ordered clusters; *D*_*i*_ values are coefficients of lateral diffusion in the bilayers; *i* = DPPC, DUPC; and *S*_*z*_ is the orientational order parameter for Martini bonds, averaged over the hydrocarbon chains in the ordered clusters.

**Table 2:** The Properties of DPPC: DUPC: Cholesterol 7:7:6 Bilayers

*T*,*K*	*A*_*L*_, nm^2^	*A*_*L*_,ordered nm^2^	*A*_*L*_,disord nm^2^	*A*_DPPC_, nm^2^	*A*_DUPC_, nm^2^	*A*_CHOL_, nm^2^	*C*_DPPC_	*C*_DUPC_	*C*_CHOL_	*D*_DPPC_, 10^7^ cm^2^/s	*D*_DUPC_, 10^7^ cm^2^/s	*S*_*z*_	Phase
360	0.580 ± 0.003	0.525 ± 0.006	0.610 ± 0.005	0.623 ± 0.006	0.690 ± 0.013	0.310 ± 0.005	0.56 ± 0.01	0.10 ± 0.01	0.34 ± 0.01	3.3 ± 0.1	4.1 ± 0.2	0.33 ± 0.03	Ld
340	0.559 ± 0.002	0.496 ± 0.007	0.597 ± 0.005	0.593 ± 0.007	0.662 ± 0.012	0.296 ± 0.005	0.57 ± 0.01	0.08 ± 0.01	0.35 ± 0.01	2.0 ±0.1	2.6 ± 0.1	0.37 ± 0.02	Ld
320	0.533 ± 0.002	0.459 ± 0.007	0.586 ± 0.006	0.553 ± 0.006	0.625 ± 0.017	0.282 ± 0.005	0.57 ± 0.01	0.07 ± 0.01	0.36 ± 0.01	1.1 ± 0.2	1.5 ± 0.1	0.42 ± 0.03	Ld
310	0.520 ± 0.002	0.436 ± 0.006	0.601 ± 0.008	0.532 ± 0.005	0.612 ± 0.017	0.274 ± 0.003	0.58 ± 0.01	0.04 ± 0.01	0.38 ± 0.01	0.6 ± 0.1	1.3 ± 0.1	0.51 ± 0.03	Lo + Ld
300	0.510 ± 0.005	0.425 ± 0.005	0.600 ± 0.007	0.519 ± 0.006	0.606 ± 0.025	0.269 ± 0.004	0.59 ± 0.01	0.02 ± 0.01	0.39 ± 0.01	0.4 ± 0.1	1.2 ± 0.1	0.60 ± 0.06	Lo + Ld
290	0.506 ± 0.003	0.421 ± 0.004	0.602 ± 0.007	0.516 ± 0.004	0.600 ± 0.019	0.270 ± 0.003	0.59 ± 0.01	0.02 ± 0.01	0.39 ± 0.01	0.15 ± 0.01	0.90 ± 0.02	0.67 ± 0.04	Lo + Ld
280	0.491 ± 0.002	0.407 ±0.002	0.588 ± 0.004	0.500 ± 0.002	0.571 ± 0.018	0.262 ± 0.003	0.59 ± 0.01	0.02 ± 0.01	0.39 ± 0.01	0.08 ± 0.02	0.65 ± 0.01	0.68 ± 0.04	Lo + Ld
270	0.481 ± 0.004	0.398 ± 0.003	0.585 ± 0.007	0.490 ± 0.004	0.544 ± 0.021	0.257 ± 0.003	0.59 ± 0.01	0.01 ± 0.01	0.40 ± 0.01	0.02 ± 0.01	0.50 ± 0.07	0.72 ± 0.02	Lo + Ld

Data for the small DPPC/DUPC/cholesterol 7:7:6 bilayer forming Ld-Lo phases. Here AL is the average area per lipid in the bilayer; AL, ordered, in the ordered clusters; AL, disord, in the remaining disordered part; Ai values are the areas per lipid components in the ordered clusters; Ci values are the concentration of lipid components in the ordered clusters; Di values are coefficients of lateral diffusion in the bilayers; i = DPPC, DUPC, and cholesterol; and Sz is the orientational order parameter for Martini bonds, averaged over the hydrocarbon chains in the ordered clusters.

The binary mixture of 3:2 DPPC/DUPC (Fig. 1) forms the L*α* phase at 340 K. In this state, the bilayer is not homogeneous and contains small clusters of the two components. These clusters are manifestations of composition fluctuations. With decreasing temperature, the bilayer becomes more heterogeneous as the composition fluctuations grow. Domains of the gel phase appear in the surrounding L*α* phase at 280 K. A detailed view of the coexisting gel-L*α* phases is given in Fig. S1 *a*. The highly ordered gel phase contrasts with the disordered L*α* phase. Phase separation in this mixture occurs below the melting temperature of the saturated lipid (*T*_*m*_ ∼ 295 K in Martini (43)); the decrease in transition temperature (the temperature at which gel phase appears, defining fluidus boundary) results from significant fraction of DUPC. This phase behavior is qualitatively similar to the DPPC-DOPC phase diagram, in which gel-L*α* phase coexistence is observed between 303 and 266 K (44). The segregation of the saturated and unsaturated lipids is driven by unfavorable interactions between the saturated and unsaturated lipid chains, which become more unfavorable as the saturated lipid transforms into the gel state (45). Transition to the gel phase is evident from an abrupt change of the average areas per lipid components (Table 1), typical for a first-order phase transition. The transition is also characterized by significant changes in the lipid lateral diffusion coefficients and the chain orientational order. Near the transition point (at 290 and 285 K), composition fluctuations become large in extent. The main phase transition in lipid bilayers is accompanied by strong fluctuations, being in vicinity to a critical point (46, 47).

The ternary mixture of 7:7:6 DPPC/DUPC/cholesterol (Fig. 2) forms the Ld phase at 340 K. It contains small clusters (enriched either in DPPC and cholesterol or in DUPC), which increase in size at 320 K. At 310 K, the bilayer separates into Lo and Ld phases. In experimental phase diagrams of DPPC/DOPC/cholesterol mixtures, the liquid-liquid coexistence is observed between ∼303 and ∼283 K, but this interval differs depending on the method (23, 48). Phase separation is induced by preferential interactions between the saturated lipid and cholesterol, leading to their segregation from the unsaturated lipid (45). A detailed view on the coexistence of the Lo and Ld phases is presented in Fig. S1 *b*. It shows that the phases noticeably differ in order, and the bilayer surface is bent at the phase boundary (see below). In contrast to the L*α*/gel mixture, strong composition fluctuations are absent; the average areas per lipid, diffusion coefficients, and chain orientational order vary gradually as the temperature decreases (Table 2). In the absence of abrupt changes in these properties, the exact phase state of the bilayer can be determined from additional analysis.

**Figure 3:**
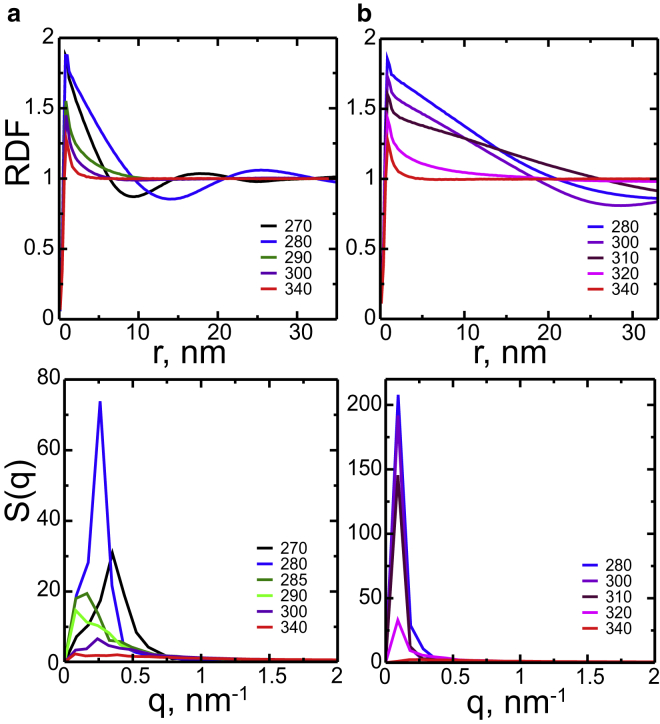
Lateral structure of the bilayers: 2D RDFs (*top panel*) and 2D structure factors (*bottom panel*), for the DPPC/DUPC 3:2 (*a*) and DPPC/DUPC/cholesterol 7:7:6 (*b*) large bilayers at different temperatures (K) are shown. RDFs and structure factors are calculated for the COMs of DUPC in the same leaflet. To see this figure in color, go online.

Composition fluctuations can be distinguished from domains of coexisting phases by analyzing the in-plane (2D) RDF (Fig. 3, *top panel*). For one-phase bilayers, the RDFs decay exponentially as the correlations in density are short range. As the temperature approaches the transition temperature, decay of RDFs occurs on a larger scale, in particular in the 3:2 DPPC/DUPC mixture at 285 and 290 K, where strong composition fluctuations are observed. For phase-separated bilayers, the RDFs decay is linear due to long-range density correlations in the domains (41, 49). Linear decay is followed by periodic undulations, which correspond to the variation of densities of the lipid components between the two coexisting phases.

Fluctuations can be also compared to domains by analyzing the 2D structure factors (Fig. 3, *bottom panel*). Molecular centers of mass of the unsaturated lipid were used as the scattering centers. Note that the resolution of the structure factors in simulations is limited by the inverse simulation box size (∼1/80 nm^−1^). The structure factors show large peaks in phase-separated bilayers. These peaks result from periodic variations of density in the coexisting phases, and correspond to a radial average of the domain spacing. In the L*α*-gel mixture, the peak is shifted toward larger wave vectors at 270 K (*q* ∼ 0.35 nm^−1^) compared to 280 K (*q* ∼ 0.26 nm^−1^) as the domains become smaller forming a thin network. Interestingly, strong composition fluctuations at 285 and 290 K manifest as intermediate peaks at similar wave vectors (*q* ∼ 0.16 nm^−1^). They result from strong correlations in space on large length scales comparable to domain size (10 nm). Note that in the Ld-Lo mixture, the peaks of the structure factors are located at smaller wave vectors (*q* ∼ 0.09 nm^−1^), as the coexisting phases span the simulation box.

To quantify composition fluctuations, we calculated the correlation lengths and times in one-phase bilayers (Fig. S2). The correlation lengths inform on the characteristic sizes of the clusters, and the correlation times on their characteristic lifetimes. As the temperature decreases and approaches the transition point, the correlation lengths increase as the fluctuations become stronger. The correlation times are expected to increase with increasing correlation length, but the data have a significant statistical uncertainty. The calculated correlation lengths and times are of the order of nanometers and nanoseconds, respectively. Nanoscale values are expected as the spatial extent of fluctuations is limited by the simulation box size (tens of nanometers).

To analyze the bilayer dynamics, we also calculated the 2D density maps averaged over different time intervals of 100 ns, 1 and 9 *μ*s (Figs. S3 and S4). Domains of coexisting phases manifest as large regions (tens of nanometers) with enrichment in density that are persistent, having lifetimes over 10 *μ*s. Composition fluctuations result in small regions (several nanometers) of density enrichment that are present on short timescales but average-out on the microsecond timescale. Strong fluctuations near the transition point are still present in 1 *μ*s but average-out in 9 *μ*s.

### Ordered clusters

**Figure 4:**
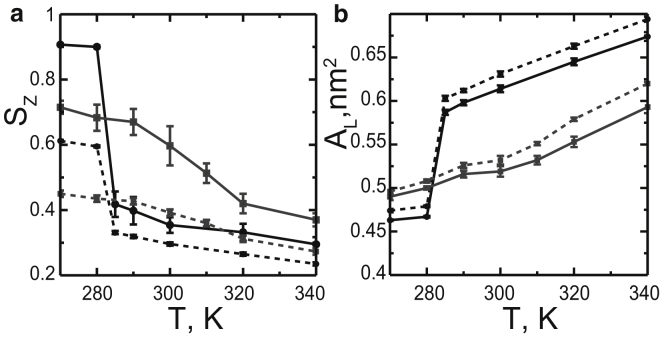
Chain orientational order parameter (*a*) and area per DPPC lipid (*b*) in the DPPC/DUPC 3:2 mixture (*black*) and the DPPC/DUPC/cholesterol 7:7:6 mixture (*gray*) are shown; solid lines correspond to the ordered clusters (see Methods), dashed lines to the averages in the bilayers.

To investigate the properties of composition fluctuations and domains of coexisting phases, we analyzed lipid clusters in each leaflet (see Methods). We clustered the sites with an increased local concentration of DPPC (and cholesterol in the ternary mixture), compared to their average concentration in the bilayer. Fig. 4 shows that these clusters are more ordered as they have a higher orientational order of lipid chains and smaller areas per lipid components compared to the bilayer averages (see also Tables 1 and 2). We thus call them the “ordered clusters”; they represent domains of the gel or Lo phase in phase-separated bilayers, and composition fluctuations in one-phase bilayers.

To assess the evolution of the demixing and phase transformations in the bilayers, we calculated the area fraction of the ordered clusters as the function of simulation time (Figs. S5 and S6). In the two-phase region at lower temperatures, the area fraction increases and reaches a plateau within several microseconds. This corresponds to formation and growth of domains of the new phase. Further transformations are related to domain merger, but this is a slow process that requires much longer simulation time to sample. In one phase at high temperatures, the clusters corresponding to composition fluctuations form and disappear; their area fraction fluctuates, but does not evolve on the simulation timescale.

The area fraction of the ordered clusters increases with decreasing temperature; domains of the more-ordered phase have a larger area fraction than fluctuations; the area fraction of Lo phase is in reasonable agreement with experimental data (27, 50). In the 3:2 DPPC/DUPC mixture, the gel phase contains mainly DPPC. In experiments on DPPC/DOPC mixtures, the gel phase has a higher fraction of DOPC (44). The area per lipid in the gel phase is almost equal to that of pure DPPC bilayers in the gel phase (0.479 nm^2^ in experiments (51) and 0.465 nm^2^ simulations with Martini model); formation of the gel phase leads to an expected drop of the diffusion coefficient by ∼2 orders of magnitude (43). In the 7:7:6 DPPC/DUPC/cholesterol mixture, the concentration of DPPC and cholesterol in the Lo domains is nearly constant (∼0.59 and 0.39, respectively). These values are comparable to previous simulations (52, 53) and are in qualitative agreement with experiments on DPPC/DOPC/cholesterol mixtures (∼0.53 of DPPC and ∼0.32–0.42 cholesterol (54)). The concentration of cholesterol in Lo phase in experiments decreases with increasing temperature, but is almost constant in our simulations. The concentration of DUPC in Lo phase (∼2%) is lower than that of DOPC in experiments. The average area per lipid in the Lo phase is in good agreement with experimental value of ∼0.44 nm^2^at 288 K (27). Based on diffusion coefficients, order parameters and areas per lipid, the Lo phase becomes substantially more ordered with decreasing temperature.

We then used the ordered clusters to analyze the differences between composition fluctuations and domains of coexisting phases. The bilayer phase state (i.e., one versus two phases) was established based on the combination of 2D RDFs (linear versus exponential decay, see above), areas per lipid, order parameters, and diffusion coefficients.

**Figure 5:**
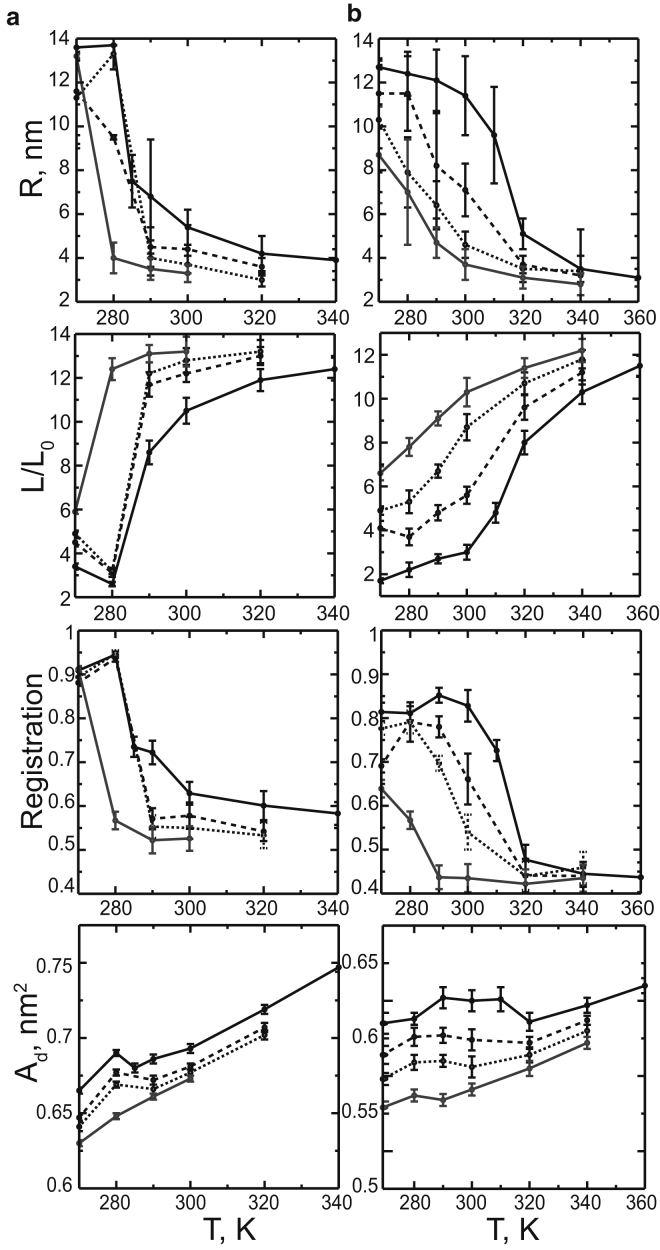
Differences in properties between domains and fluctuations for the DPPC/DUPC 3:2 (*a*) and DPPC/DUPC/cholesterol 7:7:6 (*b*) small bilayers are shown. The molar percentage of PUPC substituting DUPC is shown as follows: 0%, 20%, 30%, 40% in (*a*) or 0%, 14%, 29%, 43% in (*b*) as solid black, dashed black, dotted black, and solid gray, respectively. The horizontal panels show the following properties as a function of temperature, *T*: *R*, radius of the ordered clusters; *L*, boundary length of the ordered clusters (normalized by the perimeter of a circular cluster of the same area); overlap (registration) of the ordered clusters between the two leaflets; and *A*_*d*_, average area per lipid, in the disordered clusters (composition fluctuations or domains of the more disordered phase, L*α* or Ld).

We found that fluctuations and domains noticeably differ in several ways (see Fig. 5). The cluster radius increases with decreasing temperature, and changes significantly on phase separation. Note that due to the limited simulation box size, the radii of both fluctuations and domains lie on the nanoscale, and it is not possible to estimate the final size of the domains (i.e., nano- versus macro-). The boundary length is significantly larger for composition fluctuations compared to domains, in agreement with previous findings (41, 53). Here the boundary length was normalized by the perimeter of the circular cluster of the same area (a perfectly round cluster would thus have the boundary length of unity). Values much larger than unity indicate that fluctuations have a rough, irregular shape compared to domains.

The overlap (registration) between the two leaflets also differs significantly for domains and composition fluctuations (Fig. 5). The overlap was quantified as the area fraction of the clusters aligned between the two leaflets. Complete overlap and complete antiregistration are expected to have values of 1 and 0, respectively. The overlap of uncorrelated clusters is expected to be between 0.57 and 0.50, as the area fraction of the ordered clusters in each leaflet lies between 0.31 (at higher temperatures) and 0.46 (at lower temperatures) (55). Therefore, interleaflet correlation of composition fluctuations can be considered negligible, except for strong fluctuations in the 3:2 DPPC/DUPC mixture at 285 and 290 K (overlap > 0.7). In contrast, domains of coexisting phases overlap substantially. These differences in overlap between domains and fluctuations is consistent with the interleaflet 2D RDFs (see Fig. S7) and in agreement with previous simulations (41, 53). Earlier simulations also showed that domain overlap depends on the thickness mismatch between the Lo and Ld phases, and increases with domain size (56). Macroscopic domains in symmetric bilayers are generally found in register in experimental studies (57, 58, 59).

**Figure 6:**
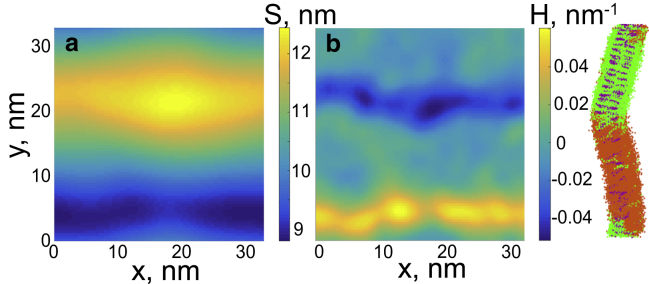
Curvature of DPPC/DUPC/cholesterol 7:7:6 bilayers at 280 K. (*a*) *S*, surface profile and (*b*) *H*, mean curvature are shown as functions of *x* and *y* coordinates in the bilayer plane. The right panel shows the corresponding side view of the bilayer; color scheme as in Fig. 2. To see this figure in color, go online.

In our simulations, overlap of Lo domains is smaller than of gel domains. Recent theoretical model predicts that incomplete registration of domains minimizes the deformation energy at the domain boundary, which reduces the line tension (60). This model assumes zero spontaneous curvatures of the leaflets. In our simulations, bilayers with the Ld-Lo phase coexistence are nonflat (see Fig. 6) and have alternating regions of negative and positive curvature (with mean curvature ∼0.05 nm^−1^). Curved symmetric bilayers with coexisting Ld and Lo phases were previously observed in simulations with the Martini model (56). The Lo phase in monolayers of similar composition in the Martini model has a negative curvature (∼−0.06 nm^−1^) (42, 61). This is in agreement with experimental studies that suggest that the Lo phase has a negative spontaneous curvature, mainly resulting from negative intrinsic curvature of cholesterol (28, 62). As the direction of bending is the opposite for the two leaflets, spontaneous curvature could not develop if the Lo domains overlapped completely. We hypothesize that incomplete overlap of Lo domains allows bending of symmetric bilayers having nonzero spontaneous curvature of the leaflets.

### Effect of the hybrid lipid

**Figure 7:**
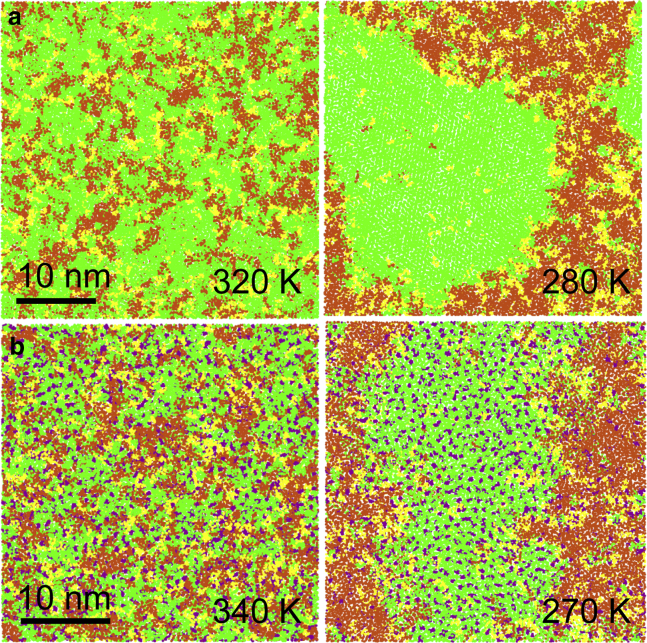
Phase behavior of the DPPC/DUPC 3:2 (*a*) and DPPC/DUPC/cholesterol 7:7:6 (*b*) small bilayers at selected temperatures, with 30 and 29% of DUPC substituted by PUPC, respectively. View from top on the upper leaflet. DPPC is shown in green, DUPC in orange, PUPC in yellow, cholesterol in purple; water not shown. To see this figure in color, go online.

We then substituted a fraction of the unsaturated lipid DUPC by the hybrid lipid PUPC in both mixtures (see Methods for details). Characteristic snapshots of the phase behavior for selected compositions are shown in Fig. 7. A summary of all simulations is given in Tables S1 and S2.

The hybrid lipid generally induces mixing in both the L*α*-gel and the Ld-Lo bilayers, i.e., the phase transition temperature decreases (see Tables 1 and 2; Tables S1 and S2). This behavior agrees with theoretical predictions (21, 63) and experimental observations (29). The distribution of PUPC is correlated with DUPC and inversely correlated with DPPC (see 2D RDFs in Figs. S8 and S9). These correlations are weak and the distribution of the hybrid lipid is nearly uniform in both mixtures in one-phase state. Approximately one-half of PUPC partitions into the ordered clusters, but only a small fraction of PUPC is present in the gel domains (see Tables S1 and S2).

The properties of composition fluctuations and domains of coexisting phases are altered by the hybrid lipid. In the L*α*-gel mixture, strong composition fluctuations near the transition temperature are suppressed. Above the transition point (290 K at 20 and 30% of PUPC, and 280 K at 40% PUPC), the 2D RDFs decay on short scales (quantified by the correlation length, see below), and the peaks on the structure factor are small (compare Fig. 3, *bottom panel*, and Fig. S10). In the Ld-Lo mixture, domains of the Lo phase appear more dynamic and disordered, and their boundary becomes more irregular (see below). The concentration in the Lo phase decreases somewhat for DPPC and more noticeably for cholesterol.

With increasing concentration of the hybrid lipid, we observe the following trends in both mixtures. The correlation lengths of fluctuations (Fig. S2, *top panel*) decrease in agreement with theoretical predictions (63). The correlation times (Fig. S2, *bottom panel*) approximately remain unchanged (given large statistical uncertainties). In other words, smaller fluctuations have longer lifetimes, which agrees qualitatively with recent theoretical predictions (64). Similar to the correlation length, the average radius of the ordered clusters decreases (Fig. 5), which is in qualitative agreement with experimental studies (where domain sizes decrease from micro- to nanoscale) (29, 30). In addition, the overlap of the clusters between the leaflets (Fig. 5) becomes smaller, as was reported earlier in simulations (35, 41).

The boundary length of domains and fluctuations (Fig. 5) increases with increasing the concentration of the hybrid lipid. Theoretical model suggest that the hybrid lipid preferentially partitions at the phase boundary, reduces the line tension and thus favors domains of smaller size (21, 65). In this model, the saturated chain of the hybrid lipid faces the saturated lipids enriched in the ordered phase, and the unsaturated chain faces the unsaturated lipids in the disordered phase. This alignment reduces the packing incompatibility and hydrophobic mismatch between the two phases, which lowers the free energy at the boundary. Previous simulations reported a small increase of the concentration of the hybrid lipid at the phase boundary (57, 66). In our simulations, the hybrid lipid does not show preferential partitioning to the boundary; its concentration at the boundary is comparable to or less than the average is the bilayer (see Tables S1 and S2).

Notably, the hybrid lipid reduces the mismatch between the coexisting phases by diluting them, which indirectly reduces the line tension at the boundary (65). This dilution effect is stronger in the disordered clusters (corresponding to L*α* or Ld), where the area per lipid decreases substantially with increasing concentration of PUPC at all temperatures, and in particular in the Ld-Lo mixture (Fig. 5, *bottom panel*). The area changes are the opposite but less pronounced in the ordered clusters (see Tables S1 and S2). The disordered clusters thus become more ordered and more comparable in properties to the ordered clusters. Similar changes for the Ld/Lo phase coexistence with varying concentration of the hybrid lipid were observed in recent experimental studies (28) and simulations (41). Besides, the area per lipid in the Ld phase changes nonmonotonically with temperature. The area per lipid in Ld phase was found to decrease with increasing temperature in recent experimental studies (27), and was correlated with increasing concentration of cholesterol in the Ld phase. We observe only a small increase of cholesterol concentration in the Ld phase with temperature at a constant concentration of PUPC. Yet as the concentration of PUPC increases, the concentration of cholesterol in the disordered clusters also increases.

## Discussion

We investigated nanoscale domains of coexisting phases and composition fluctuations in lipid bilayers. Molecular dynamics simulations with the CG Martini model were used. Simple lipid mixtures of a saturated lipid, an unsaturated lipid, and cholesterol separated into either L*α*/gel or the Ld/Lo phases. The properties of domains and fluctuations along the bilayer transition from a two-phase to a one-phase state were characterized. The transition was induced by varying the temperature and lipid composition, where the unsaturated lipid was partially substituted by the hybrid lipid.

The CG simulations show the changes in phase behavior of lipid bilayers with temperature. As the temperature decreases and crosses the transition temperature, the bilayers transform from a mixed state to a phase-separated state. The observed changes in phase behavior with temperature are qualitative and are weaker as the Martini force field underestimates the temperature dependence of the bilayer properties. This is due to a partial substitution of entropic interactions by enthalpic interactions, resulting from a partial loss of degrees of freedom intrinsic to coarse-graining. Importantly, the changes of phase behavior with temperature in the Martini model have the correct trend, and phase transformation and separation in lipid membranes have been previously reproduced in many simulations (33, 34).

The phase behavior of our mixtures can be qualitatively compared to experimental phase diagrams. As the Martini model has reduced chemical detail compared to atomistic models, it gives the same molecular representation for lipids with small differences, e.g., in the length and unsaturation of hydrocarbon chains. Combined with weaker temperature dependence mentioned above and potential offset in the main phase transition temperatures of individual lipids (34), quantitative comparison is challenging in coarse-grained simulations in general. Atomistic simulations are expected to avoid some of these issues, but sampling the length and timescales that are necessary to reproduce phase coexistence remains the major challenge. In fact, no phase separation has been simulated from random mixtures in atomistic models to date. With currently available methods and resources, Martini simulations provide a reasonable computational approach to study phase behavior of lipid membranes.

Our simulations inform on nanoscale lateral organization in lipid membranes at high spatial and temporal resolution in the absence of probes or labels. This detailed information can complement experimental studies, where characterization of the properties of nanoscale structures is complicated by their small sizes and short lifetimes.

The mixed state represents a heterogeneous liquid phase, where the degree of heterogeneity increases with decreasing temperature. At high temperatures, lipid mixtures are almost random, containing only small dynamic clusters. They have characteristic sizes of nanometers and lifetimes of nanoseconds. These clusters are enriched in selected components (DPPC, cholesterol) forming a more ordered phase (gel or Lo) at lower temperatures. These clusters are manifestations of density and composition fluctuations driven by more favorable interactions between specific lipid types (45). The clusters have the properties of the host phase (L*α* or Ld), i.e., the fluctuations are homo-phase in nature, as they are associated with the same phase state (67). Yet the clusters are more ordered compared to the surrounding liquid phase, based on the lateral density (inversed area per lipid) and chain orientational order. This local compositional demixing and ordering leads to dynamic heterogeneity with local structure (22). As the transition temperature is approached from above, the characteristic size and lifetime of the clusters increases, i.e., the fluctuations cover tens of nanometers and persist on microsecond timescales. Interestingly, in the L*α*/gel mixture the fluctuations are comparable in size to domains and lead to strong density correlations in space that show on the in-plane structure factor.

Below the phase transition temperature, bilayers form domains of coexisting phases. Whereas fluctuations are transient, domains of coexisting phases are static, persistent in time and space. They differ from fluctuations in the concentration of the components, areas per lipid, order parameters, and diffusion coefficients. Interestingly, these properties changed abruptly in the 3:2 DPPC/DUPC mixture upon formation of the gel phase, but continuously in the 7:7:6 DPPC/DUPC/cholesterol mixture upon formation of the Lo phase. In addition, the Ld/Lo bilayers have persistent regions of positive and negative curvature that we believe develop due to negative spontaneous curvature of the Lo domains in each leaflet at incomplete overlap of domains between the two leaflets.

Model membranes of a saturated and an unsaturated lipid and cholesterol, despite their simplicity, have a phase behavior consistent with cell membranes, as has been demonstrated in giant plasma membrane vesicles (GPMVs) (68, 69). GPMVs form one liquid phase at physiological temperature and liquid-liquid coexistence of Lo-like and Ld-like phases at room temperatures. This is a remarkable finding, allowing us to reduce the compositional complexity: while containing multiple components, cell membrane appear to limit their phase behavior to only two liquid phases. Separation into Lo and Ld phases in simple lipid mixtures can be considered a common model for lateral organization in cell membranes (12).

In our model membrane simulations, we found significant differences between composition fluctuations and domains of coexisting phases. First, domains have distinct properties of the second phase, whereas fluctuations have the properties of the host phase. Second, composition fluctuations generally have negligible overlap between the two leaflets, whereas the overlap of domains of coexisting phases is substantial. Overlap is likely required for functional coupling between the inner and outer leaflets in cell membranes. Finally, the boundary length of composition fluctuations is substantially longer than that of domains of coexisting phases, producing much more irregular, rough morphology. Nanoscale domains and fluctuations are both potential candidates for rafts in cell membranes. These results provide important insights that can be used to determine the nature of rafts or—more generally—of nanoscale lateral heterogeneity in cell membranes.

Our simulations show systematic changes in the bilayer phase behavior upon partial substitution of the unsaturated lipid by the hybrid lipid. The hybrid lipid reduces the phase transition temperature and alters the properties of both composition fluctuations and domains of coexisting phases. Its effects can be summarized as follows: a reduction of the size of Lo domains, a reduction of the correlation length of fluctuations in both the L*α*/gel and Ld/Lo mixtures, a decrease in overlap of domains and fluctuations between leaflets, and an increase of the boundary length of domains and fluctuation in both mixtures. Preferential partitioning of the hybrid lipid to the boundary is not observed. Notably, an increase of the boundary length is related to dilution of the coexisting phases: ordering of the disordered clusters (domains), which thus become more similar in properties to the ordered clusters (domains). Interestingly, the Ld phase in GMPVs is much more ordered than in model membranes, which can be attributed to the majority of the hybrid lipids in the former (70). This leads to a smaller difference in order between the Ld and Lo phases in plasma membranes, and to a higher partitioning and activity of selected proteins in the Lo phase. In addition, an increase of the boundary length is indicative of a decrease of the line tension. Line tension has been found to produce abrupt changes of domain sizes in recent experiments on model bilayers (71). Taken together, these results point to a potential biological role of hybrid lipids of tuning the lateral sorting of proteins (and lipids) by adjusting the order and size of the coexisting domains.

## Conclusions

We simulated simple lipid bilayers with liquid-liquid and solid-liquid phase coexistence. A gradual transition from a two-phase to a one-phase state was induced by raising the temperature or adding a hybrid lipid. Along the transition, domains of coexisting phases change to composition fluctuations with local ordering and compositional demixing. Nanoscale fluctuations and domains differ in several key properties, which can be used to understand the nature of nanoscale lateral organization (rafts) in cell membranes. Hybrid lipids reduce the difference between the coexisting phases, which suggest a potential biological role in tuning the order and size of domains in biological membranes.

## Author Contributions

S.B. and D.P.T. designed the simulations. S.B. performed the simulations. S.B. and D.R. performed analyses. S.B. and D.P.T. wrote the manuscript.

## Supporting Material

Document S1. Figs. S1–S10 and Tables S1 and S2

Document S2. Article plus Supporting Material
