# Supplementary material for: Composition Fluctuations in Lipid Bilayers
Source: Biophys J. 2017 Dec 19;113(12):2750–61. doi: 10.1016/j.bpj.2017.10.009 (PMC5770567; doi:10.1016/j.bpj.2017.10.009)
Supplement: Document S1. Figs. S1–S10 and Tables S1 and S2 [file mmc1.pdf]

Biophysical Journal, Volume *113*

Supplemental Information

Composition Fluctuations in Lipid Bilayers

Svetlana Baoukina, Dmitri Rozmanov, and D. Peter Tieleman

Composition fluctuations in lipid bilayers

S. Baoukina, D. Rozmanov, and D. P. Tieleman.

Supporting information

Tables

Table S1. Bilayer properties in the Lα-gel mixture with hybrid lipid.

a.

T,K	A_L, ordered nm^2	A_{DPPC}, nm^2	A_{DUPC}, nm^2	A_{PUPC}, nm^2	C_{DPPC}	C_{DUPC}	C_{PUPC}	D_{DPPC}, 10^7 cm^2/s	D_{DUPC}, 10^7 cm^2/s	D_{PUPC}, 10^7 cm^2/s	S_z	C_b	Phase
320	0.649 ±0.004	0.641 ±0.005	0.714 ±0.016	0.677 ±0.018	0.87 ±0.01	0.09 ±0.01	0.04 ±0.01	3.1 ±0.1	3.2 ±0.1	3.3 ±0.3	0.33 ±0.01	0.05 ±0.01	Lα
300	0.622 ±0.004	0.616 ±0.004	0.684 ±0.012	0.647 ±0.019	0.88 ±0.01	0.08 ±0.01	0.04 ±0.01	1.7 ±0.1	1.8 ±0.1	1.8 ±0.3	0.38 ±0.02	0.06 ±0.01	Lα
290	0.605 ±0.004	0.598 ±0.004	0.673 ±0.014	0.635 ±0.017	0.89 ±0.01	0.07 ±0.01	0.04 ±0.01	1.5 ±0.1	1.4 ±0.1	1.4 ±0.1	0.39 ±0.03	0.06 ±0.01	Lα
280	0.467 ±0.001	0.466 ±0.001	0.585 ±0.04	0.502 ±0.015	0.97 ±0.01	0.01 ±0.01	0.02 ±0.01	0.08 ±0.03	0.82 ±0.06	0.66 ±0.03	0.89 ±0.01	0.09 ±0.01	gel+Lα
270	0.463 ±0.001	0.463 ±0.001	0.532 ±0.018	0.482 ±0.007	0.97 ±0.01	0.01 ±0.01	0.02 ±0.01	0.02 ±0.01	0.37 ±0.03	0.23 ±0.03	0.91 ±0.01	0.09 ±0.01	gel+Lα

b.

T,K	A_L, ordered nm^2	A_{DPPC}, nm^2	A_{DUPC}, nm^2	A_{PUPC}, nm^2	C_{DPPC}	C_{DUPC}	C_{PUPC}	D_{DPPC}, 10^7 cm^2/s	D_{DUPC}, 10^7 cm^2/s	D_{PUPC}, 10^7 cm^2/s	S_z	C_b	Phase
320	0.651 ±0.003	0.644 ±0.003	0.711 ±0.016	0.683 ±0.014	0.87 ±0.01	0.08 ±0.01	0.06 ±0.01	3.0 ±0.1	3.1 ±0.1	3.0 ±0.1	0.33 ±0.02	0.07 ±0.01	Lα
300	0.621 ±0.004	0.615 ±0.004	0.682 ±0.014	0.650 ±0.019	0.87 ±0.01	0.07 ±0.01	0.06 ±0.01	1.9 ±0.1	2.0 ±0.1	2.0 ±0.1	0.37 ±0.03	0.08 ±0.01	Lα
290	0.606 ±0.003	0.600 ±0.003	0.672 ±0.012	0.631 ±0.017	0.88 ±0.01	0.06 ±0.01	0.05 ±0.01	1.3 ±0.1	1.3 ±0.1	1.3 ±0.2	0.40 ±0.02	0.08 ±0.01	Lα
280	0.465 ±0.001	0.464 ±0.001	0.556 ±0.05	0.493 ±0.009	0.97 ±0.01	0.01 ±0.01	0.02 ±0.01	0.10 ±0.01	0.76 ±0.01	0.8 ±0.1	0.90 ±0.05	0.11 ±0.01	gel+Lα
270	0.462 ±0.001	0.461 ±0.001	0.520 ±0.042	0.476 ±0.007	0.96 ±0.01	0.01 ±0.01	0.03 ±0.01	0.03 ±0.01	0.47 ±0.06	0.28 ±0.04	0.89 ±0.08	0.11 ±0.01	gel+Lα

c.

T,K	A_L, ordered nm^2	A_{DPPC}, nm^2	A_{DUPC}, nm^2	A_{PUPC}, nm^2	C_{DPPC}	C_{DUPC}	C_{PUPC}	D_{DPPC}, 10^7 cm^2/s	D_{DUPC}, 10^7 cm^2/s	D_{PUPC}, 10^7 cm^2/s	S_z	C_b	Phase
300	0.621 ±0.003	0.614 ±0.003	0.681 ±0.015	0.643 ±0.014	0.86 ±0.01	0.06 ±0.01	0.08 ±0.01	1.7 ±0.1	1.8 ±0.1	1.9 ±0.1	0.37 ±0.02	0.10 ±0.01	Lα
290	0.608 ±0.003	0.602 ±0.004	0.666 ±0.015	0.636 ±0.018	0.87 ±0.01	0.06 ±0.01	0.08 ±0.01	1.4 ±0.1	1.5 ±0.2	1.4 ±0.1	0.40 ±0.02	0.10 ±0.01	Lα
280	0.592 ±0.004	0.585 ±0.004	0.663 ±0.014	0.621 ±0.012	0.88 ±0.01	0.05 ±0.01	0.07 ±0.01	1.2 ±0.1	1.2 ±0.1	1.2 ±0.1	0.42 ±0.02	0.10 ±0.01	Lα
270	0.461 ±0.001	0.460 ±0.001	0.552 ±0.057	0.473 ±0.004	0.95 ±0.01	0.00 ±0.01	0.05 ±0.01	0.03 ±0.01	0.42 ±0.01	0.30 ±0.04	0.92 ±0.01	0.12 ±0.01	gel+Lα

Data for small DPPC:DUPC 3:2 bilayers with 20 %(a), 30%(b), and 40%(c) of DUPC substituted by PUPC. Here A_L is the average area per lipid in the ordered clusters, A_i are the areas per lipid components and C_i are the concentration of lipid components in the ordered clusters; D_i are coefficients of lateral diffusion in the bilayers; i=DPPC, DUPC, PUPC; S_z is the orientational order parameter for Martini bonds, averaged over the hydrocarbon chains in the ordered clusters; C_b is concentration (molar ratio) of the hybrid lipid PUPC at the boundary; the concentration of the hybrid lipid in the bulk is 0.08, 0.12, and 0.16 for the three considered compositions.

Table S2. Bilayer properties in the Ld-Lo mixture with hybrid lipid.

a.

T,K	A_L, ordered nm^2	A_{DPPC}, nm^2	A_{DUPC}, nm^2	A_{PUPC}, nm^2	A_{CHOL}, nm^2	C_{DPPC}	C_{DUPC}	C_{PUPC}	C_{CHOL}	D_{DPPC}, 10^7 cm^2/s	D_{DUPC}, 10^7 cm^2/s	D_{PUPC}, 10^7 cm^2/s	S_z	C_b	Phase
340	0.500 ±0.007	0.592 ±0.007	0.658 ±0.016	0.625 ±0.025	0.297 ±0.004	0.56 ±0.01	0.08 ±0.01	0.03 ±0.01	0.33 ±0.01	2.3 ±0.01	2.6 ±0.2	2.3 ±0.2	0.38 ±0.02	0.05 ±0.01	Ld
320	0.469 ±0.005	0.560 ±0.006	0.633 ±0.013	0.590 ±0.019	0.285 ±0.004	0.57 ±0.01	0.06 ±0.01	0.02 ±0.01	0.35 ±0.01	1.2 ±0.01	1.6 ±0.1	1.6 ±0.1	0.43 ±0.03	0.06 ±0.01	Ld
300	0.429 ±0.006	0.519 ±0.007	0.603 ±0.021	0.553 ±0.020	0.269 ±0.004	0.58 ±0.01	0.03 ±0.01	0.03 ±0.01	0.37 ±0.01	0.46 ±0.02	0.89 ±0.02	0.84 ±0.02	0.51 ±0.04	0.06 ±0.01	Lo+Ld
290	0.427 ±0.004	0.518 ±0.004	0.598 ±0.018	0.558 ±0.016	0.271 ±0.003	0.58 ±0.01	0.02 ±0.01	0.02 ±0.01	0.38 ±0.01	0.25 ±0.01	0.76 ±0.02	0.70 ±0.04	0.57 ±0.06	0.09 ±0.01	Lo+Ld
280	0.416 ±0.004	0.506 ±0.005	0.573 ±0.027	0.529 ±0.012	0.266 ±0.003	0.58 ±0.01	0.02 ±0.01	0.02 ±0.01	0.38 ±0.01	0.12 ±0.02	0.64 ±0.02	0.44 ±009	0.67 ±0.03	0.09 ±0.01	Lo+Ld
270	0.413 ±0.003	0.501 ±0.002	0.563 ±0.023	0.522 ±0.015	0.265 ±0.002	0.59 ±0.01	0.01 ±0.01	0.02 ±0.01	0.38 ±0.01	0.07 ±0.01	0.38 ±0.01	0.26 ±0.02	0.70 ±0.04	0.09 ±0.01	Lo+Ld

b.

T,K	A_L, ordered nm^2	A_{DPPC}, nm^2	A_{DUPC}, nm^2	A_{PUPC}, nm^2	A_{CHOL}, nm^2	C_{DPPC}	C_{DUPC}	C_{PUPC}	C_{CHOL}	D_{DPPC}, 10^7 cm^2/s	D_{DUPC}, 10^7 cm^2/s	D_{PUPC}, 10^7 cm^2/s	S_z	C_b	Phase
340	0.505 ±0.006	0.595 ±0.008	0.660 ±0.019	0.636 ±0.020	0.299 ±0.004	0.56 ±0.01	0.07 ±0.01	0.05 ±0.01	0.33 ±0.01	2.3 ±0.1	2.5 ±0.2	2.3 ±0.1	0.38 ±0.03	0.06 ±0.01	Ld
320	0.476 ±0.006	0.565 ±0.005	0.631 ±0.014	0.601 ±0.014	0.288 ±0.003	0.56 ±0.01	0.05 ±0.01	0.05 ±0.01	0.34 ±0.01	1.4 ±0.1	1.7 ±0.1	1.7 ±0.1	0.42 ±0.04	0.06 ±0.01	Ld
300	0.446 ±0.005	0.533 ±0.004	0.604 ±0.013	0.569 ±0.011	0.276 ±0.003	0.57 ±0.01	0.03 ±0.01	0.05 ±0.01	0.35 ±0.01	0.7 ±0.1	0.88 ±0.07	0.89 ±0.02	0.49 ±0.03	0.07 ±0.01	Lo+Ld
290	0.436 ±0.003	0.523 ±0.003	0.597 ±0.017	0.557 ±0.013	0.274 ±0.002	0.57 ±0.01	0.02 ±0.01	0.04 ±0.01	0.36 ±0.01	0.33 ±0.04	0.63 ±0.01	0.61 ±0.05	0.55 ±0.04	0.08 ±0.01	Lo+Ld
280	0.427 ±0.004	0.514 ±0.003	0.577 ±0.026	0.545 ±0.011	0.272 ±0.002	0.58 ±0.01	0.01 ±0.01	0.04 ±0.01	0.37 ±0.01	0.19 ±0.02	0.61 ±0.02	0.41 ±0.09	0.61 ±0.05	0.09 ±0.01	Lo+Ld
270	0.417 ±0.002	0.502 ±0.002	0.567 ±0.032	0.530 ±0.009	0.266 ±0.002	0.59 ±0.01	0.01 ±0.01	0.04 ±0.01	0.37 ±0.01	0.07 ±0.02	0.44 ±0.02	0.26 ±0.02	0.69 ±0.04	0.10 ±0.01	Lo+Ld

c.

T,K	A_L, ordered nm^2	A_{DPPC}, nm^2	A_{DUPC}, nm^2	A_{PUPC}, nm^2	A_{CHOL}, nm^2	C_{DPPC}	C_{DUPC}	C_{PUPC}	C_{CHOL}	D_{DPPC}, 10^7 cm^2/s	D_{DUPC}, 10^7 cm^2/s	D_{PUPC}, 10^7 cm^2/s	S_z	C_b	Phase
340	0.505 ±0.008	0.593 ±0.007	0.662 ±0.021	0.629 ±0.013	0.300 ±0.004	0.55 ±0.01	0.05 ±0.01	0.07 ±0.01	0.32 ±0.01	2.5 ±0.1	2.8 ±0.3	2.8 ±0.2	0.38 ±0.02	0.08 ±0.01	Ld
320	0.479 ±0.006	0.564 ±0.006	0.633 ±0.019	0.597 ±0.017	0.288 ±0.004	0.56 ±0.01	0.04 ±0.01	0.07 ±0.01	0.33 ±0.01	1.3 ±0.1	1.7 ±0.1	1.4 ±0.1	0.45 ±0.02	0.09 ±0.01	Ld
300	0.453 ±0.006	0.538 ±0.005	0.606 ±0.021	0.568 ±0.014	0.278 ±0.003	0.57 ±0.01	0.03 ±0.01	0.06 ±0.01	0.34 ±0.01	0.71 ±0.02	0.94 ±0.09	0.93 ±0.01	0.49 ±0.02	0.09 ±0.01	Ld
290	0.441 ±0.004	0.523 ±0.003	0.589 ±0.021	0.554 ±0.012	0.274 ±0.003	0.57 ±0.01	0.02 ±0.01	0.06 ±0.01	0.34 ±0.01	0.41 ±0.02	0.60 ±0.07	0.56 ±0.02	0.53 ±0.03	0.09 ±0.01	Ld
280	0.430 ±0.003	0.513 ±0.003	0.576 ±0.024	0.540 ±0.009	0.270 ±0.003	0.58 ±0.01	0.02 ±0.01	0.05 ±0.01	0.35 ±0.01	0.19 ±0.01	0.43 ±0.01	0.40 ±0.04	0.60 ±0.03	0.11 ±0.01	Lo+Ld
270	0.420 ±0.003	0.503 ±0.040	0.562 ±0.028	0.527 ±0.009	0.266 ±0.003	0.58 ±0.01	0.01 ±0.01	0.06 ±0.01	0.35 ±0.01	0.10 ±0.01	0.35 ±0.04	0.26 ±0.06	0.65 ±0.06	0.11 ±0.01	Lo+Ld

Data for small DPPC:DUPC:cholesterol 7:7:6 bilayers with 14% (a), 29% (b), and 43% (c) of DUPC substituted by PUPC. Here A_L is the average area per lipid in the ordered clusters, A_i are the areas per lipid components and C_i are the concentration of lipid components in the ordered clusters; D_i are coefficients of lateral diffusion in the bilayers; i=DPPC, DUPC, PUPC, cholesterol; S_z is the orientational order parameter for Martini bonds, averaged over the hydrocarbon chains in the ordered clusters; C_b is concentration (molar ratio) of the hybrid lipid PUPC at the boundary; the concentration of the hybrid lipid in the bulk is 0.05, 0.10, and 0.15 for the three considered compositions.

Figures

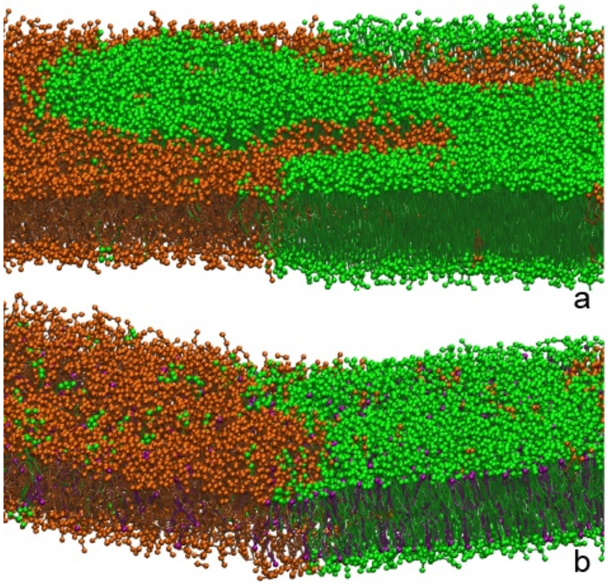

Figure S1. Detailed view on the coexistence of gel-Lα (a) and Lo-Ld phases (b). The DPPC: DUPC 3:2 mixture at 280 K and the DPPC: DUPC: cholesterol 7:7:6 mixture at 290 K are shown.

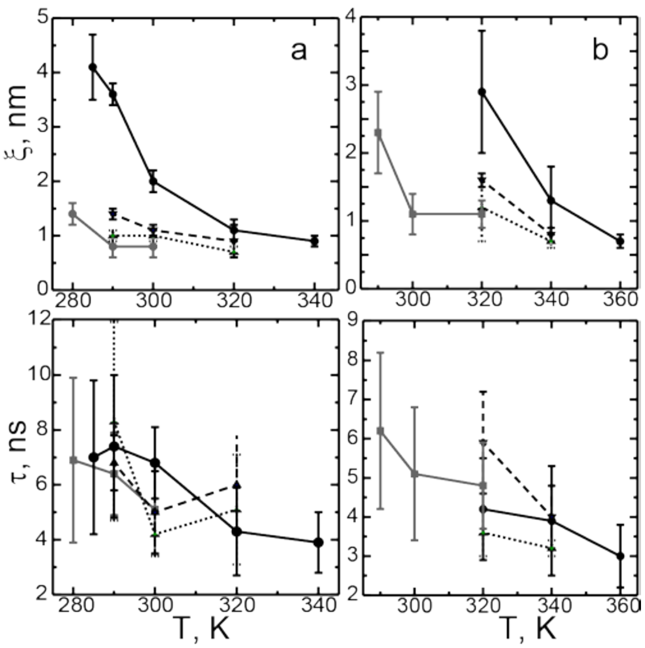

Figure S2. Correlation length (top panel) and time (bottom panel) for the DPPC: DUPC 3:2 (left panel a) and DPPC: DUPC: cholesterol 7:7:6 (right panel b) small bilayers. Different molar % of PUPC substituting DUPC are shown as follows: 0%, 20%, 30%, 40% in (a,c) or 0%, 14%, 29%, 43% in (b,d) as solid black, dashed black, dotted black, and solid grey, respectively.

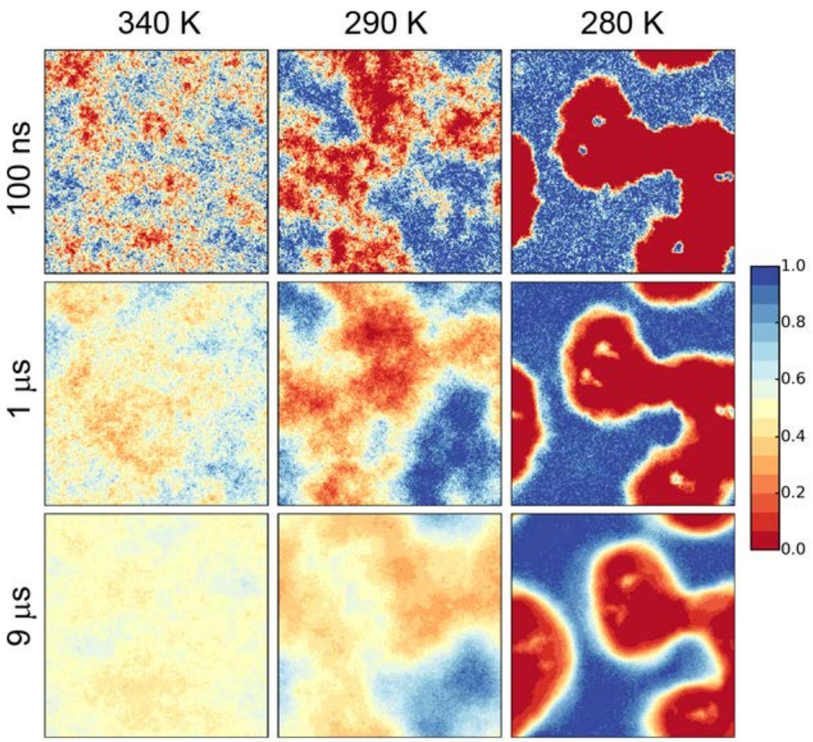

Figure S3. 2D density maps for the binary mixture of DPPC:DUPC 3:2 at selected temperatures averaged over the last 100 ns, 1 μs and 9 μs of simulations. The density is calculated for DUPC lipids in one of the leaflets and is normalized by the double average DUPC density, i.e. the value of 0.5 corresponds to the average DUPC density in the leaflet.

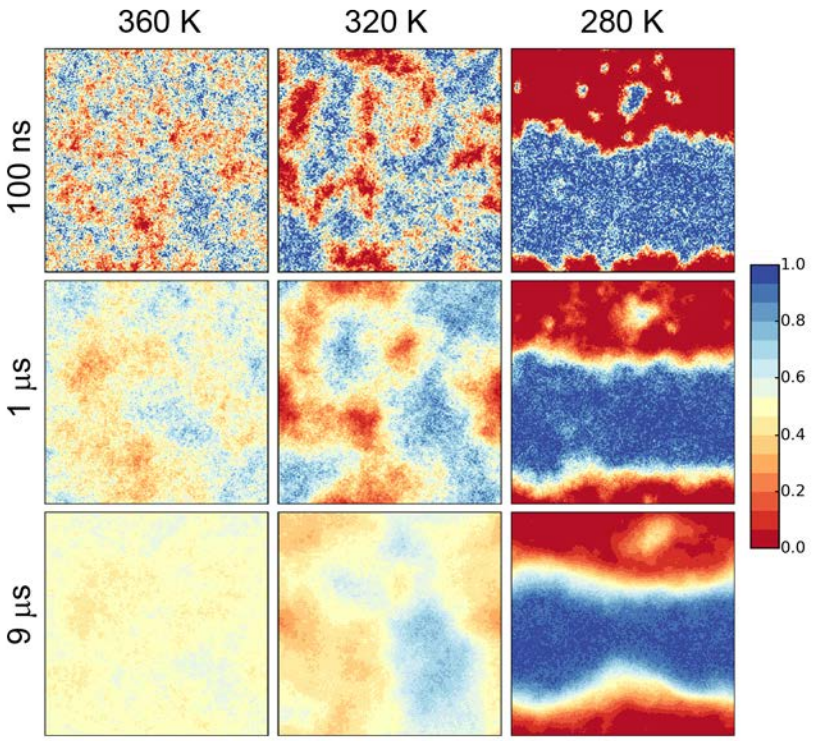

Figure S4. 2D density maps for the ternary mixture of DPPC:DUPC:CHOL 7:7:6 at selected temperatures averaged over the last 100 ns, 1 μs and 9 μs of simulations. The density is calculated as in Figure S6.

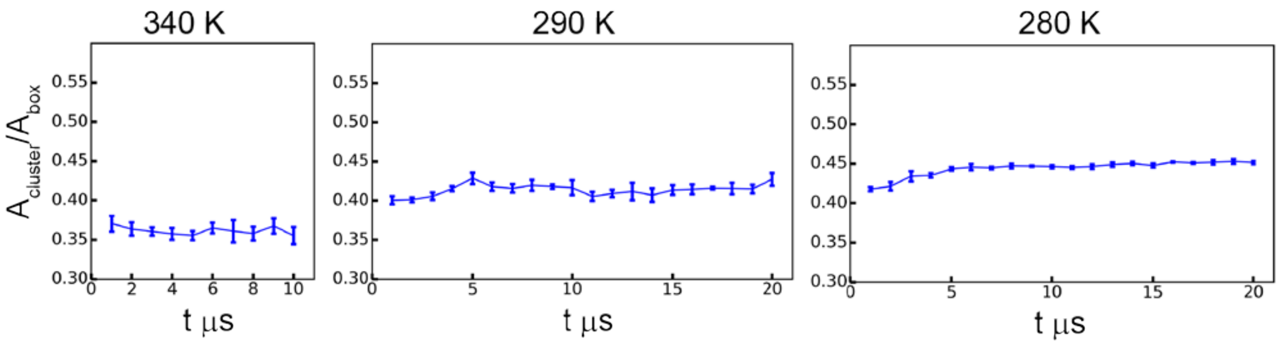

Figure S5. The area of the ordered clusters, normalized by the simulation box area, as a function of time at selected temperatures for the DPPC: DUPC 3:2 small bilayers.

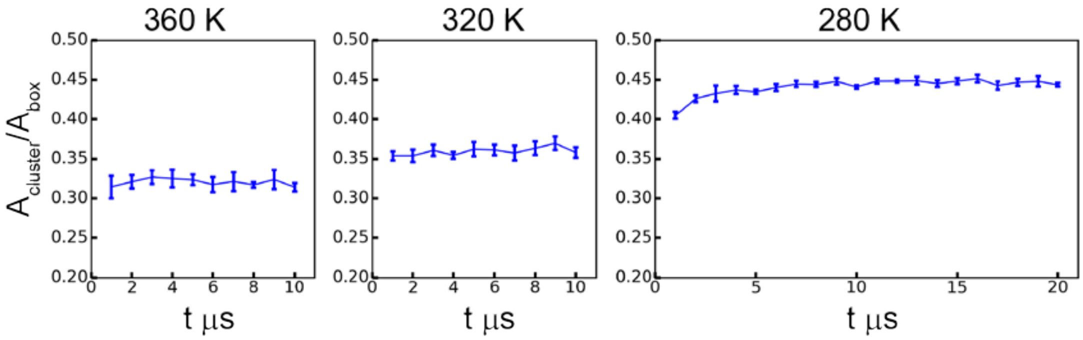

Figure S6. The area of the ordered clusters, normalized by the simulation box area, as a function of time at selected temperatures for the DPPC: DUPC: cholesterol 7:7:6 small bilayers.

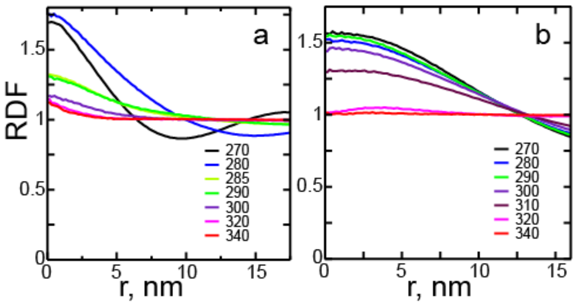

Figure S7. Inter-leaflet 2D RDFs for the DPPC: DUPC 3:2 (a) and DPPC: DUPC: cholesterol 7:7:6 (b) small bilayers at different temperatures (K). RDFs are calculated for the centers of mass of DUPC lipids in the opposite leaflets.

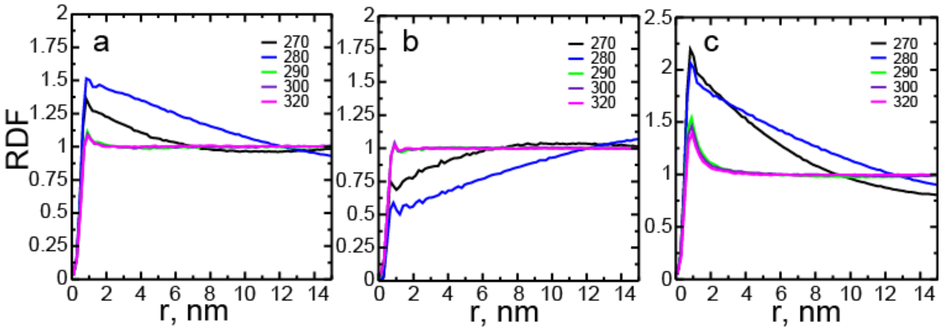

Figure S8 2D RDFs for the DPPC: DUPC 3:2 small bilayer with 30% of DUPC substituted by PUPC. The distributions are calculated for the molecular centers of mass in the same leaflet; PUPC-DUPC (a) and PUPC-DPPC (b), and DUPC-DUPC (c) distributions are shown.

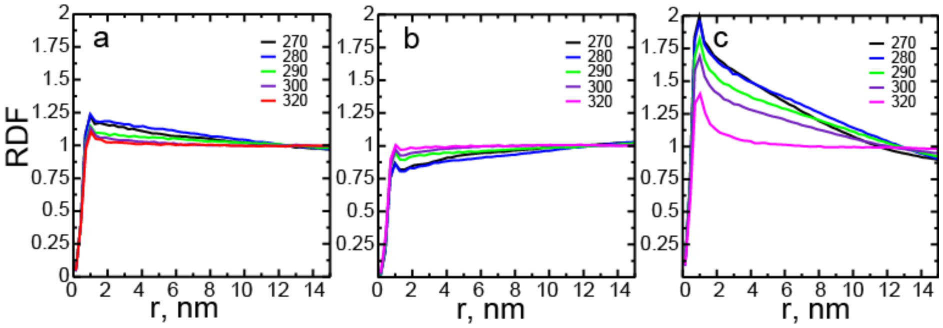

Figure S9 2D RDFs for the DPPC: DUPC: cholesterol 7:7:6 small bilayers with 29% DUPC substituted by PUPC at different temperatures (K). The distributions are calculated for the molecular centers of mass in the same leaflet; PUPC-DUPC (a) and PUPC-DPPC (b), and DUPC-DUPC (c) distributions are shown.

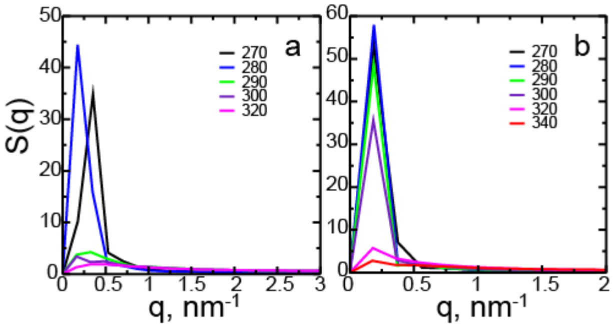

Figure S10. 2D structure factors for the DPPC: DUPC 3:2 small bilayer with 20% PUPC substituting DUPC (a), and for the DPPC: DUPC: cholesterol 7:7:6 small bilayer with 14% PUPC substituting DUPC (b) at different temperatures (K). Structure factors are calculated for the molecular centers of mass of DUPC.
